# Genetic analysis of tolerance to Boron toxicity in the legume *Medicago truncatula*

**DOI:** 10.1186/1471-2229-13-54

**Published:** 2013-03-27

**Authors:** Paul Bogacki, David M Peck, Ramakrishnan M Nair, Jake Howie, Klaus H Oldach

**Affiliations:** 1 South Australian Research and Development Institute, Plant Genomics Centre, Waite Campus, Urrbrae, SA, 5064, Australia; 2 University of Adelaide, Waite Campus, Urrbrae, SA, 5064, Australia; 3 AVRDC - The World Vegetable Center, ICRISAT Campus, Patancheru 502 324, Hyderabad, Andhra Pradesh, India

**Keywords:** *Medicago truncatula*, Abiotic stress, Legume, Boron toxicity tolerance, Genetic analysis, *MtNIP3*, Gene cluster, Differential expression

## Abstract

**Background:**

*Medicago truncatula* Gaertn. (barrel medic) is cultivated as a pasture legume for its high protein content and ability to improve soils through nitrogen fixation. Toxic concentrations of the micronutrient Boron (B) in agricultural soils hamper the production of cereal and leguminous crops. In cereals, the genetic analysis of B tolerance has led to the development of molecular selection tools to introgress and maintain the B tolerance trait in breeding lines. There is a comparable need for selection tools in legumes that grow on these toxic soils, often in rotation with cereals.

**Results:**

Genetic variation for B tolerance in *Medicago truncatula* was utilised to generate two F_2_ populations from crosses between tolerant and intolerant parents. Phenotyping under B stress revealed a close correlation between B tolerance and biomass production and a segregation ratio explained by a single dominant locus. *M*. *truncatula* homologues of the Arabidopsis major intrinsic protein (MIP) gene *AtNIP5;1* and the efflux-type transporter gene *AtBOR1*, both known for B transport, were identified and nearby molecular markers screened across F_2_ lines to verify linkage with the B-tolerant phenotype. Most (95%) of the phenotypic variation could be explained by the SSR markers *h2_6e22a* and *h2_21b19a*, which flank a cluster of five predicted *MIP* genes on chromosome 4. Three CAPS markers (*MtBtol-1,-2*,*-3*) were developed to dissect the region further. Expression analysis of the five predicted *MIPs* indicated that only *MtNIP3* was expressed when leaf tissue and roots were assessed. *MtNIP3* showed low and equal expression in the roots of tolerant and intolerant lines but a 4-fold higher expression level in the leaves of B-tolerant cultivars. The expression profile correlates closely with the B concentration measured in the leaves and roots of tolerant and intolerant plants. Whereas no significant difference in B concentration exists between roots of tolerant and intolerant plants, the B concentration in the leaves of tolerant plants is less than half that of intolerant plants, which further supports *MtNIP3* as the best candidate for the tolerance trait-defining gene in *Medicago truncatula*.

**Conclusion:**

The close linkage of the *MtNIP3* locus to B toxicity tolerance provides a source of molecular selection tools to pasture breeding programs. The economical importance of the locus warrants further investigation of the individual members of the *MIP* gene cluster in other pasture and in grain legumes.

## Background

*Medicago truncatula* Gaertn. (barrel medic; *Fabaceae*), native to the Mediterranean region, is a self-regenerating annual pasture species grown on over 4.5 million hectares in the dryland cereal/livestock zones in southern Australia [1]. *M. truncatula* is adapted to a wide range of soils from sandy loams to clays and prefers neutral to alkaline soils (pH (water) > 6.5). When grown in rotation with cereal crops their ability to fix atmospheric nitrogen contributes significantly to the nitrogen balance of following crops. This lessens the need for inorganic nitrogen fertilisers and hence increases economic returns to producers and benefits the environment [2,3]. Additionally as a non-host of many important cereal diseases, they are able to provide a valuable disease-break from pathogens such as Take-all (*Gaeumannomyces graminis*), crown rot (*Fusarium graminearum*) and cereal cyst nematode (*Heterodera avenae*) [4]. Together with *Lotus japonicus*, *M*. *truncatula* is also a model organism for legume biology with an international consortium providing researchers with numerous genetic resources including two drafts of the sequenced genome and genetic maps (http://www.medicagohapmap.org). Its relatively small genome size (ca. 500 Mbp) and high levels of synteny to major crop legumes such as pea (*Pisum sativum*) and alfalfa (*M*. *sativa*) [5] make it an ideal system to conduct molecular genetics analyses on biotic and abiotic stresses affecting legumes.

A major problem facing *M*. *truncatula* and other legumes in arid world regions such as southern Australia, northern Africa, and the Mediterranean is the presence of phytotoxic levels of Boron (B) in the soil which can significantly impact on seed yield and quality [6]. Because remediation of B-toxic soils is impractical [7], investigators have looked to improve plant tolerance to B. In cereal crop species such as wheat and barley, genetic variation for B tolerance has led to the identification of quantitative trait loci (QTL) linked to B toxicity tolerance [8], and more recently to the genes underlying these QTL [9-11]. This has facilitated the development of closely linked molecular markers that can be used in breeding programs to improve the tolerance of wheat and barley grown in areas where B toxicity is a problem. In *M*. *truncatula*, genetic variation for B toxicity tolerance among different cultivars had earlier been reported [6] but so far has not been exploited to identify the gene(s) or genetic regions controlling this trait.

Recent studies in barley and Arabidopsis offer clues as to what genes may control B toxicity tolerance in *M*. *truncatula*. These studies have implicated the involvement of genes encoding one of two types of proteins involved in B uptake and translocation: (1) efflux-type B transporters or (2) members of the major intrinsic protein (MIP) family. The first efflux-type B transporter gene identified was the Arabidopsis *AtBOR1* gene [12]. Although *AtBOR1* conferred tolerance to plants under B deficient conditions, its homologue in barley, *HvBot1* (identical to *HvBOR2*), enabled plants to tolerate high levels of B [9,11]. The over-expression of an *AtBOR1* paralog, *AtBOR4*, in transgenic Arabidopsis plants also increased their tolerance to high B levels [13]. *AtNIP5;1* was the first B transporter gene identified that encodes a member of the MIP family [14]. MIPs, also known as aquaporins, are channels for water and/or small non-charged molecules [15] and are comprised of four subfamilies; the nodulin 26-like intrinsic proteins (NIPs), the plasma membrane intrinsic proteins (PIPs), the tonoplast intrinsic proteins (TIPs), and the small basic intrinsic proteins (SIPs). *AtNIP5;1* encodes a NIP subfamily member, and like *AtBOR1*, was shown to be associated with B deficiency tolerance, with AtNIP5;1 functioning as a boric acid channel required for the efficient uptake of B in roots [14]. In contrast, a recently discovered *NIP* gene in barley, *HvNIP2;1*, was found to be an important determinant of B toxicity tolerance [10], with the authors proposing that B toxicity tolerance is mediated by reduced expression of *HvNIP2;1* to limit B uptake as well as by increased expression of *HvBot1* to remove B from roots and sensitive tissues. A MIP subfamily member, *AtTIP5;1* was also recently described conferring tolerance to B toxicity when over-expressed in transgenic Arabidopsis plants [16].

In this study, a candidate gene approach combined with population genetics and phenotypic analysis was used to determine the molecular basis of B tolerance in *M. truncatula.* We report the identification of a homologous *AtNIP5;1* gene, *MtNIP3*, in *M*. *truncatula* associated with B toxicity tolerance. A gene-based marker derived from *MtNIP3*, named *MtBtol-1*, and further markers co-segregated with B toxicity tolerance in two F_2_ populations. In addition, gene expression of *MtNIP3* was up-regulated in the leaves of respective B-tolerant parental lines. This information warrants further characterisation of *MtNIP3* as a candidate gene for B tolerance and provides gene-based markers as molecular tools to introgress and maintain the tolerance trait in Medicago breeding programs.

## Results

### Segregation analysis

The inheritance results of the phenotypic data are presented in Table 1. The segregation ratio for the B toxicity tolerance trait has a significant fit (*p* = 0.05) to the expected 3:1 (tolerant vs. intolerant) ratio for a single dominant locus, which is true for both F_2_ populations. F_2_ plants of the population *tap* × Paraggio (intolerant x tolerant) had leaf symptom scores ranging from 0 to 4, whereas in *tap* × Caliph the scores ranged from 0 to 5. Relative to these symptom ranges in *t*xP and *t*xC, plants with a score of 0 and 1 in *t*xP and a score of 0 to 2 in *t*xC were considered as B tolerant.

**Table 1:** **Phenotypic segregation of the B toxicity tolerance trait in the F**_**2 **_**populations *tap *x Paraggio and *tap *x Caliph**

**Population**	**No. of plants phenotyped**	**No. of B-tolerant F**_**2 **_**plants**	**No. of B-intolerant F**_**2 **_**plants**	**Segregation ratio**	**χ**^**2**^
*tap* x Paraggio	316	247	69	3 : 1	0.882
*tap* x Caliph	313	228	85	3 : 1	0.406

### B accumulation in root and leaf tissue

**Figure 1:**
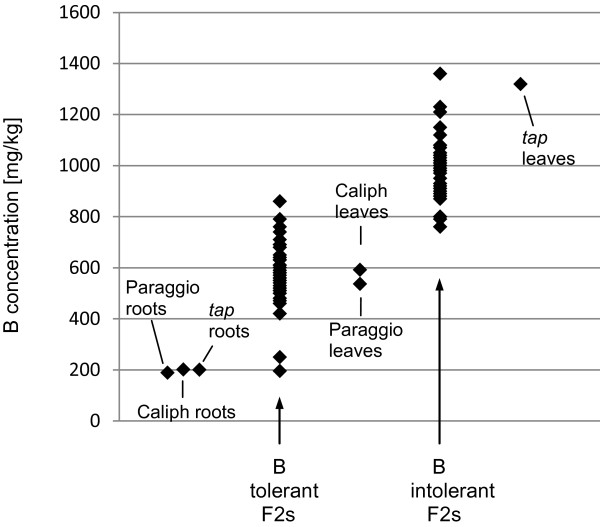
**Distribution of B concentrations in tolerant and intolerant *M.t. *lines after 3 weeks of excess B.** The amount of B was quantified in leaves of tolerant and intolerant F_2_ lines of both populations and the two tolerant parental lines Paraggio and Caliph and the intolerant parent *tap*. ICP data were generated on 35–40 F_2_ lines of the tolerant and intolerant subsets of both populations, 4–8 repeats of leaf and 4 repeats of root samples for each parent.

Initially, subsets of 39 and 36 F_2_ lines from the *t*xP and *t*xC populations, respectively, were selected to screen for potential molecular markers. The subsets consisted of lines that were phenotyped after three weeks of high B treatment and had clear tolerant (0 or 1) or intolerant (4 or 5) scores. ICP analysis was used to (a) quantify the amount of B accumulating in the leaf, and (b) verify the correlation between B levels and phenotypic scores. Additional ICP analysis was carried out on leaves and roots of the three parental lines to quantify the B distribution in tolerant and intolerant plants. The results are summarised in Figure 1. In the *t*xP subset, the mean B concentration in leaves sampled from B-tolerant and -intolerant F_2_ lines was 559 mg/kg (range: 420–710) and 942 mg/kg (range: 760–1360), respectively. In the *t*xC subset the mean B concentration was 605 mg/kg (range: 195–860) and 1021 mg/kg (range: 870–1210). The leaves of parental lines had mean B concentrations of 536 (Paraggio), 592 (Caliph), and 1319 (*tap*) mg/kg. In contrast to the mean B concentrations in the leaves, with tolerant lines accumulating less than half the amount of B compared to the intolerant lines, mean B concentrations in the roots did not differ significantly, 189 (Paraggio), 201 (Caliph) and 200 (*tap*) mg/kg.

### Biomass production and Boron tolerance

**Figure 2:**
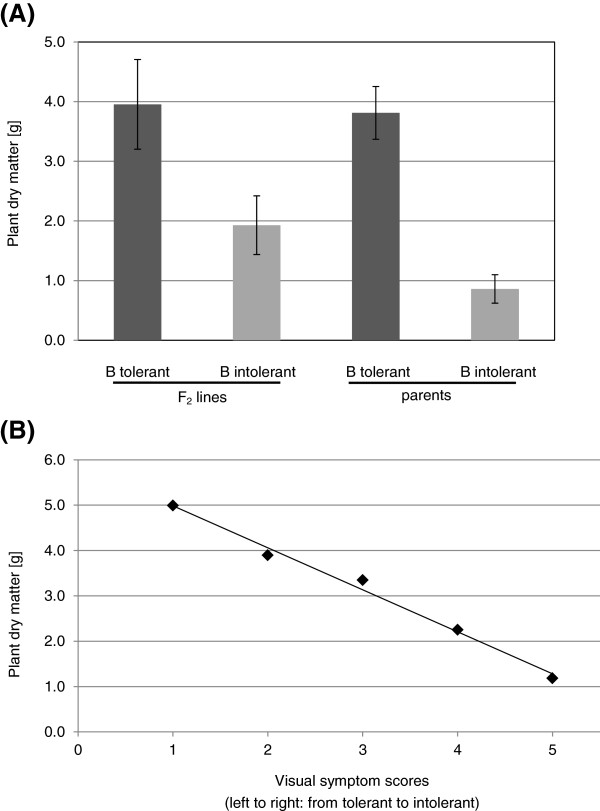
**Mean plant dry matter (DM) comparison.** (**A**) DM of tolerant and intolerant F_2_ lines is compared to tolerant and intolerant parental lines (Caliph, *tap*) of the corresponding population. Error bars represent standard deviations of the mean. (**B**) High negative correlation (−0.995) between DM and severity of the visual B stress symptom scores. n = 21, 42, 32, 30, 13 for plants with visual symptom score 1, 2, 3, 4, 5, respectively.

Breeding for B tolerance aims at maintaining yield under high B concentrations. As a proxy for the agronomic value of the B tolerance trait in pasture legumes, plant biomass or dry matter production (DM) was measured. The average DM production of the tolerant F_2_ plants and their tolerant parent, Caliph, was not significantly different, 4.0 g versus 3.8 g, respectively. In contrast, the average DM of the intolerant F_2_ lines was significantly higher than that of the intolerant parent *tap*, 1.9 g versus 0.9 g, respectively (Figure 2A). The DM and symptom scores were highly correlated with a coefficient of −0.995 (Figure 2B) indicating that the observed differences in biomass production are due to the B tolerance trait being present or absent in the individual F_2_ plants. With each increase in visual symptom score DM was reduced by approximately one gram.

### Development of molecular markers for B toxicity tolerance

The *AtBOR1* and *AtNIP5;1* cDNA sequences (GenBank: AB073713 and NM_117106) and their respective predicted protein sequences (GenBank: BAC20173 and NP_192776) were used to query the *M*. *truncatula* pseudomolecule database (version Mt3.5) available at the *M*. *truncatula* Hapmap project website (http://www.medicagohapmap.org). The best *AtBOR1* homologue, identified on chromosome 7 (BAC AC157892; GeneID: *Medtr7g110000*.1) encodes a predicted protein, which shares 78% amino acid (aa) sequence identity with AtBOR1. We developed a gene based CAPS marker for this gene but found no linkage between this Boron transporter gene and B tolerance in either of the *t*xP or *t*xC F_2_ subsets.

**Figure 3:**
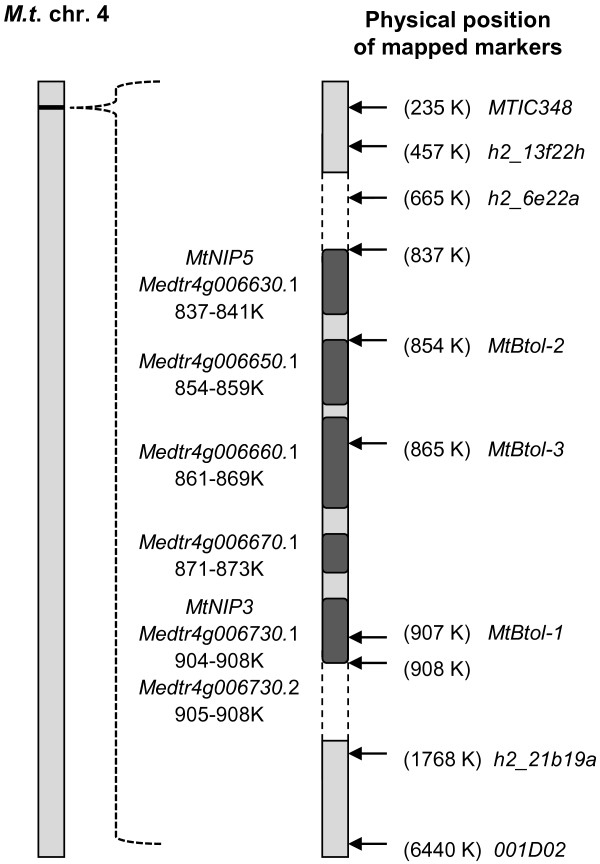
**Chromosome region linked to B tolerance.** Scheme of the genome region on linkage group 4 in *M. truncatula* (version Mt3.5) showing the physical position of the B tolerance candidate genes and markers.

Four Medicago database matches to *AtNIP5;1* were identified on chromosomes 1 (BACs AC181971 and AC225543) and 4 (BACs AC168305 and AC153003). Both chromosome 1 candidates (GeneID: *Medtr1g097840*.1 and *Medtr1g098080*.1) encode exactly the same predicted protein, which shares 69% sequence identity with AtNIP5;1, whereas the chromosome 4 candidates encode different predicted proteins with 60% (GeneID: *Medtr4g006730*.1) and 54% (GeneID: *Medtr4g006630*.1) identity to AtNIP5;1, respectively. The *Medtr4g006730*.1 protein product corresponds to the aquaporin MtNIP3 (GenBank: AY539749) identified [17] and its corresponding gene is referred to as *MtNIP3*. The two BACs on each chromosome were adjacent to one another and the physical distance between candidate genes was small – approximately 84 kb and 71 kb for chromosome 1 and 4 candidates, respectively. Two flanking SSR markers (*002G02*, *h2*_*13c11a*) located within 500 kb of the chromosome 1 candidate genes showed polymorphisms between *tap* and Paraggio but proved not to be linked to B tolerance in the subset of F_2_ lines tested. Thus, like the *AtBOR1* homologue on chromosome 7, the chromosome 1 candidate genes were ruled out from further analysis. In contrast, SSR markers flanking the *NIP* candidate genes on chromosome 4 showed strong linkage with the B tolerance phenotype. The SSR markers *MTIC348*, *h2_13f22h*, *h2_6e22a*, *h2_21b19a* and *001D02*, which flank a 6205 kb interval were polymorphic in either one or both F_2_ populations and explained 94% to 96% of the phenotypic variation (Table 2, Figure 3). Amongst these SSRs, the markers which were more distant from the 71 kb *NIP* gene cluster were less closely linked to the tolerance phenotype within the segregating populations (Table 2, Figure 3). Together with the segregation ratio characteristic of a single dominant locus, the strong linkage of these SSR makers to the tolerant phenotypes indicated that the trait-defining gene(s) reside at this 71 kb *NIP* gene locus.

**Table 2:** **Molecular markers and the phenotypic variation they explain in the two F**_**2 **_**populations studied**

**Marker**	**Marker, *Mt *chromosome**	**Primer sequence (forward and reverse, 5’ – 3’)**	**Restriction enzyme**	**Expected CAPS fragment size [bp]**		**Phenotypic variation explained [%]**	
				***tap***	**Par/Cal**	*t *x P*	*t *x C**
*MTIC348*	SSR, chr. 4	TGGAGGAGGGGTAGGATAGG ATGATGATGAGGCGGAGAAG	-	-	-	95	94
*h2_13f22h*	SSR, chr. 4	GGCTTCCTGATGCTGGTTAG ACAAGCAGGTTGGACACACA	-	-	-	95	94
***h2_6e22a***	SSR, chr. 4	CCATGGTGTCATTTGATCCT CCATGGTGTCATTTGATCCT	-	-	-	96	n.p.
***MtBtol-1***	CAPS (gene), chr. 4	CTCTTTACCTCCCCCTCCTG TTGAACTACGATCAGGGTCGTTCCTA	*ApoI*	387	170/217	96	94
***MtBtol-2***	CAPS (gene), chr. 4	TTGAACTACGATCAGGGTCGTTCCTA TGCTCCAGCACATCCAATTA	*DraI*	102/131	102/256	96	94
				256/280	411		
***MtBtol-3***	CAPS (gene), chr. 4	TCCCACTGAAAATCGATTCAA GGCCAAAGCTCATTCCATT	*MspI*	125/450	575	96	94
***h2_21b19a***	SSR, chr. 4	GGAACACCGGACACAATACC CACTTCTCAGGCACAGCAAA	-	-	-	96	n.p.
*001D02*	SSR, chr. 4	TCCCAACGCTTTTTCATTTC GAACTTGAAGAAGAACGCCG	-	-	-	94	89
*MtChr7*	CAPS, chr. 7	TTGGAGCATTTATACCAGCAA TTGTATGCATCGGAGACTGC	*NlaIII*	306	84/222	n.a.	n.a.
*002G04*	SSR, chr. 1	AGGGTCGCCTCAACTATTA TCAACACCATTTTCTCAATG	-	-	-	n.a.	n.a.
*002G02*	SSR, chr. 1	TAACGTTACTCCCTCCTCCG TCTCCAACGAAGTTCAAGGG	-	-	-	n.a.	n.a.
*h2_13c11a*	SSR, chr. 1	TCACAACCTTCTTGCACCAG TCATAACTTGACCTGCACCA	-	-	-	n.a.	n.a.

Most closely linked markers are in bold font. * *n* = 200; ** *n* = 36; n.a., not applicable; n.p., not polymorphic.

**Figure 4:**
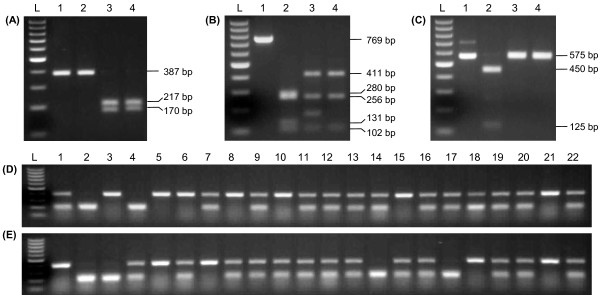
**CAPS markers linked to B toxicity tolerance.** (**A**) *MtBtol-1*; (**B**) *MtBtol-2*; (**C**) *MtBtol-3*; Lane L - DNA ladder, Lane 1 – undigested amplicon from *tap*, Lanes 2 to 4 – digested amplicons from *tap*, Caliph, and Paraggio, respectively. (**D**) and (**E**), Application of *MtBtol-1* on genomic DNA from 22 randomly selected F_2_ plants derived from the *tap* x Paraggio and *tap* x Caliph crosses, respectively, distinguishing homozygous and heterozygous F_2_ plants.

To dissect this locus, we derived CAPS markers from or closer to the *NIP* genes themselves. The *M*. *truncatula* Hapmap project gene annotations revealed that the candidate *AtNIP5;1* homologue on BAC AC153003 has a potential splice variant, with both gene products (GeneID: *Medtr4g006730*.1 and *Medtr4g006730*.2) predicted to encode NIPs. Likewise, the second chromosome 4 candidate on BAC AC168305 (GeneID: *Medtr4g006630*.1) is also predicted to encode a NIP, with the 71 kb genomic region in between the two genes (*Medtr4g006730*.1 and *Medtr4g006630*.1) containing three more potential *NIP* genes (GeneIDs: *Medtr4g006650*.1*/660*.1*/670*.1). Therefore, according to the gene annotations, the locus as a whole contains up to five predicted *NIP* genes encoding six gene products potentially involved in controlling B toxicity tolerance. Three CAPS markers, named *MtBtol-1*, *MtBtol-2* and *MtBtol-3* (Table 2, Figure 3, Figure 4A-C) were developed that were polymorphic in both *t*xP (200 F_2_ lines tested) and *t*xC (36 F_2_ lines tested) populations (Figure 4D, E) within this region and explained 96% and 94% of the phenotypic variation, respectively. *MtBtol-1* is specific to the first candidate *AtNIP5;1* homologue, *Medtr4g006730*.1*/730.*2, *MtBtol-2* is specific to the *Medtr4g006650*.1 gene which is situated 13 kb away from the second candidate *AtNIP5;1* homologue, *Medtr4g006630*.1, and *MtBtol-3* is specific to the *Medtr4g006660*.1 gene. It was not possible to design a gene specific marker for *Medtr4g006630*.1 due to its close similarity with the *Medtr4g006730*.1*/730.*2 gene.

### *NIP* gene expression

We used Q-PCR to investigate the expression profiles of the *NIP* genes in roots and shoots of the three parental lines over a 48 h period following excess B treatment. Due to the high level of coding sequence similarity between the *Medtr4g006630*.1*/730*.1*/730.*2 gene products, it was only possible to design gene specific primers for *Medtr4g006630*.1 and the splice variant *Medtr4g006730*.2. Design of gene specific primers was also hindered by the absence of information related to the untranslated regions of these gene sequences. Hence, a third primer pair, specific to all three genes, was designed to generate a cumulative expression profile of B transporter candidates. Each of the primer pairs amplified the expected sized fragment when genomic DNA was used as template, however, only the primer pair specific for all three gene products amplified clear fragments of expected size from the cDNAs of the time course experiment. Two *Medtr4g006630*.1 specific primer pairs with different annealing positions in the gene amplified no product at all whereas the *Medtr4g006730.*2 specific primers yielded a small amount of expected product corresponding to <10 copies/μL in the leaf and root tissue examined, which is at the very limit of detection for Q-PCR. Therefore, by exclusion, the cumulative expression profile that was generated for all three genes was in fact presumed to represent one, *Medtr4g006730*.1.

**Figure 5:**
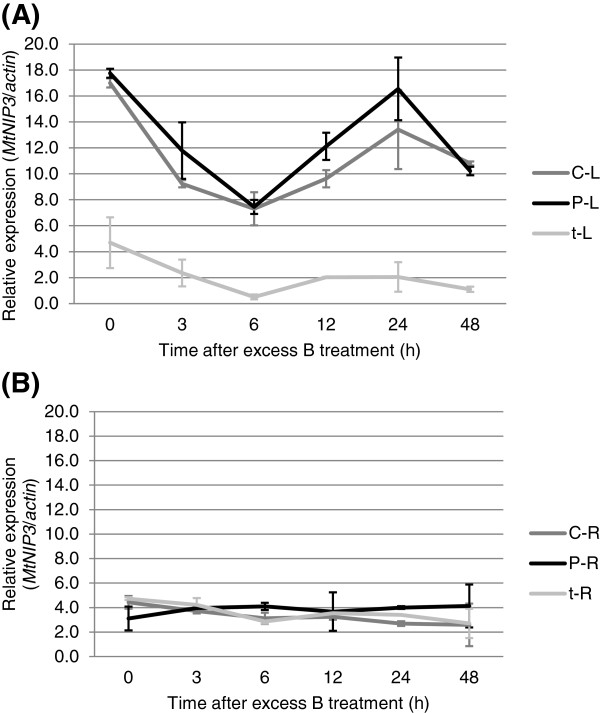
**Quantitative RT-PCR of *MtNIP3*.** (**A**) *MtNIP3* expression in Paraggio (P), Caliph (C), and *tap (t)* is regulated in the leaves (**B**) but not in roots following excess B treatment. Error bars represent standard error of the means, *n* = 2.

The expression profile for *Medtr40006730*.1 showed that the gene is constitutively expressed in the leaves, with approximately 4 times more transcripts present in both B-tolerant lines compared to the intolerant line (Figure 5A). In roots, the expression was equally low and non-differential in all three genotypes irrespective of their B tolerance phenotype. The expression level in the roots was comparable with the leaf expression of the intolerant line *tap* (Figure 5B). The stronger gene expression of *Medtr40006730.*1 in the leaves of the tolerant lines correlated with the B toxicity tolerance phenotype.

Q-PCR assays were also designed to investigate the possible involvement of the other three predicted *MIP* genes at this locus, *Medtr4g006650*.1*/660*.1*/670*.1. Again, the specificity of the primers was confirmed with the correct sized PCR fragment being amplified from DNA template. However, none of the primer pairs amplified any product on the cDNAs of the time course experiment from leaf and root tissue, suggesting no detectable expression before or after B treatment.

### Analysis of *MtNIP3* (*Medtr4g006730*.1) gene copy number

**Figure 6:**
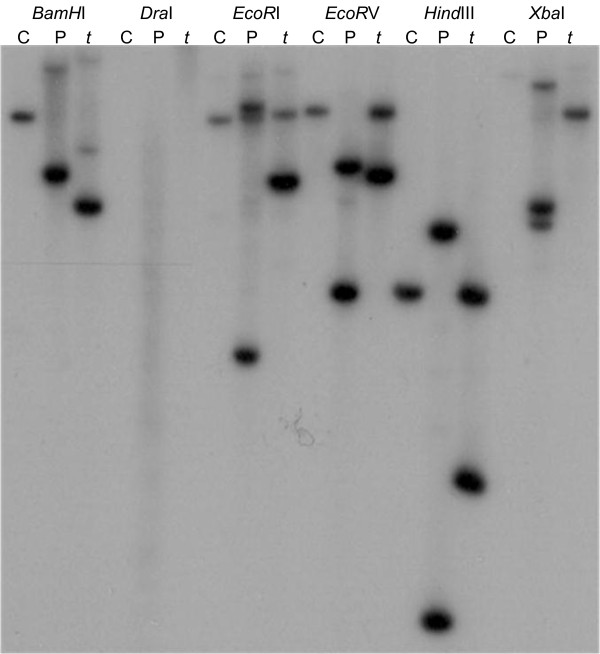
**Southern hybridisation with an *MtNIP3 *(*Medtr4g006730*.1)-specific probe in Caliph (C), Paraggio (P) and *tap *(*t*).**

We used Southern hybridisation to determine if the presence of additional *MtNIP3* gene copies in the two B-tolerant lines could account for the increased mRNA transcript levels observed for this gene in the leaves. As it was not possible to design a specific hybridisation probe for *Medtr4g006730*.1 (*MtNIP3*) due to the high level of homology it shares with *Medtr4g006630*.1 (*MtNIP5*), the designed probe would detect both genes. The resulting hybridisation revealed the presence of two gene copies in *tap* (as expected) and Paraggio, and one copy in Caliph (Figure 6). Thus, the observed increase in *MtNIP3* transcript levels in the leaves of the two B-tolerant lines was not associated with a higher gene copy number.

### *MtNIP3* coding sequence and promoter region

The *MtNIP3* coding region was sequenced from Caliph, Paraggio, and *tap* to identify potential differences in the predicted amino acid composition of the corresponding MIP protein. A 918 bp cDNA fragment was amplified from all three genotypes. None of the seven single nucleotide polymorphisms (SNPs) identified were specific to Caliph/Paraggio or *tap* (Additional file 1). The seven SNPs translated into three changes among the 305 amino acid residues of the NIP protein in the three genotypes. Again, the changes were not specific to the B-tolerant genotypes (Additional file 2).

A comparative *Medtr4g006730*.1 promoter analysis between Caliph, Paraggio, and *tap* was also performed to search for differences which could explain the variation in transcript levels observed between the B-tolerant and -intolerant lines. Sequencing of the 1.1 kb region directly upstream of the transcription start site revealed a 99% basepair identity between the three genotypes. The differences were not specific to the intolerant or the tolerant genotypes and further work would be required to address the impact of these single and dinucleotide differences on gene expression or search for potential differences further upstream.

## Discussion

In this study, a candidate gene approach was used to identify the underlying genetic loci for tolerance to B toxicity in *M*. *truncatula.* Similar to the recently reported identification of the genetic basis for herbicide tolerance in *M*. *truncatula*[18], we utilised the molecular and physiological information on B homeostasis first reported in Arabidopsis [12,14]. Although the corresponding proteins of the Arabidopsis B transporter /channel genes *AtBOR1* and *AtNIP5;1* are associated with the efficient transport of B across the plasma membrane under B deficiency [12,14], similar proteins in barley were shown to aid the plant in conditions of B excess [9-11]. Therefore, it was hypothesised that homologous genes in *M*. *truncatula* of *AtNIP5;1* and *AtBOR1* could also play a role in supporting plants to tolerate high levels of B in the soil.

Genetic variation for B toxicity tolerance was identified in the *M*. *truncatula* lines Paraggio and Caliph (tolerant, [6]) and *tap* (intolerant). Paraggio and Caliph were each crossed with *tap* to generate two F_2_ populations segregating for B tolerance. The phenotypic segregation of the B tolerance trait was measured in over 300 individuals for each of the F_2_ populations and agreed with a single dominant locus that might host a single or several closely linked tolerance genes. A very close correlation between the visual symptom scores on individual plants and their biomass production was also evident suggesting B uptake as the direct and major cause of reduced plant growth and validating the symptom scores as a valid tool to estimate plant performance. All tolerant plants analysed showed a reduced (on average ca. 50%) level of B in their leaf tissue compared to intolerant lines implying an exclusion mechanism being the underlying B tolerance mechanism in this pasture legume. An exclusion mechanism was also proposed in barley where B-tolerant cultivars showed decreased B concentrations in their leaves compared to intolerant cultivars [11,19].

With the aid of the 2nd draft (version Mt3.5) of the sequenced *M*. *truncatula* genome, candidate *AtNIP5;1* and *AtBOR1* homologues were identified on chromosomes 1, 4, and 7. A subset of highly B-tolerant and -intolerant lines from the two segregating F_2_ populations was used initially for genotyping to establish linkage between the B-tolerant phenotype and SSR markers located close to the homologous *Bor* and *NIP* genes on all three chromosomes. Only SSR markers located near two candidate *AtNIP5;1* homologues, separated by only 71 kb, on chromosome 4 showed linkages of approximately 95% to the tolerance trait in both F_2_ populations. The strong linkage was then upheld across 200 individual F_2_ lines from the *tap* x Paraggio cross which further supported that the corresponding genetic region was associated with B toxicity tolerance. We referred to one of these candidate genes as *MtNIP3* and the predicted protein as MtNIP3, which was one of six aquaporins first identified in *M*. *truncatula*[17]. The other predicted gene was referred to as *MtNIP5*.

Although both corresponding proteins encoded by *MtNIP3* and *MtNIP5* confirmed good amino acid sequence similarity to AtNIP5;1, they both showed a greater level of amino acid sequence identity to AtNIP6;1 – another MIP family member and B channel identified in Arabidopsis [20]. MtNIP3 has 75% sequence identity to AtNIP6;1 whereas MtNIP5 shares only 57% identity. Within the 71 kb interval, between *MtNIP3* and *MtNIP5*, there are up to three additional predicted *MIP* genes and a splice variant of *MtNIP3*. Fine mapping this locus with three CAPS markers derived from the actual *MIP* genes themselves or sequences nearby did not improve the linkage obtained with the two most closely linked SSR markers. However, the slight decrease in linkage for flanking SSR markers either side of this locus gave a strong indication that one or a combination of the *MIP* genes in the 71 kb interval were involved in regulating the transport of B in the plant. Therefore, as an output of the genetic analysis we identified eight molecular markers that were closely linked to the B tolerance trait, the best of which were CAPS markers *MtBtol-1*, *MtBtol-2*, and *MtBtol-3* and SSR markers *h2_6e22a* and *h2_21b19a*, which are all 95% linked. The observation that these markers explain 95% and not 100% of the phenotypic variation is expected considering that B-intolerant F_2_ plants still show a greater level of B tolerance, when quantified as DM production, than the intolerant parent (Figure 2A). Plant characteristics independent of B exclusion such as morphological differences e.g. a stronger vigour of Caliph and Paraggio over the intolerant *tap* mutant, are likely to account for the remaining 5% of phenotypic variation in these two populations. Aside from DM production, the ICP analysis clearly showed differences in the range of B levels in the leaves of the F_2_ lines from the *t*xC compared to the *t*xP population. These differences can again be explained by genotype-specific traits (e.g. maturity, morphology) that are inherited independently of the B exclusion locus.

As the chromosome 4 locus associated with *MtBtol-1* contains up to five predicted *MIP* genes, we tried to narrow down the association with B toxicity tolerance to individual genes by monitoring their expression in Caliph, Paraggio, and *tap* prior to and after treatment with excess B. Although a specific Q-PCR assay could not be designed for *MtNIP3*, by excluding the low or non-expression of its predicted splice variant and the similar gene *MtNIP5*, respectively, we deduced that *MtNIP3* was the only *MIP* gene expressed at this locus as no expression signals were detected for the remaining three *MIP* candidates when using alternative sets of primers. This meant that they were either not expressed, expressed at extremely low levels, or that the gene predictions were not correct and these genes were simply not there. Ultimately, the presence and function of *in silico* predicted genes has to be confirmed through the analysis of their gene product [21]. Most interesting was the observation that the constitutive expression of *MtNIP3* in the leaves was almost four-fold higher in both B-tolerant lines compared to the intolerant line. By contrast, there was no difference in expression levels in the roots of tolerant and intolerant lines. A similar pattern was observed with the distribution of B in the leaves and roots of the tolerant and intolerant parental lines. There was no significant difference of B in the roots but twice the B concentration in the leaves of the intolerant parent compared to that in both tolerant lines. Several mechanisms of alleviating B toxicity are being discussed, including efflux from the roots, redistribution of B in the leaves and leaching of B by rain or removing B from the plant through guttation [22]. Based on the observed B distribution in the roots and leaves we can exclude that efflux from the roots is responsible for the different levels of B tolerance in these genotypes. Nevertheless, other B tolerant medic genotypes [6] might well employ such a mechanism. Possible mechanisms could be either the redistribution of B in the leaves from sensitive symplastic to apoplastic spaces in the leaves and subsequent leaching through rain [22] and/or the removal of B via guttation [9,22,23]. After B treatment, *MtNIP3* expression was regulated in the leaves only, with the level of differential expression being 4 times higher in B-tolerant compared to intolerant lines. The fact that the two B-tolerant lines also have substantially less B in their leaves suggests that *MtNIP3* itself or homologs at the *MtNIP3* locus may function in removing B from the leaf.

Southern hybridisation with an *MtNIP3*/*MtNIP5* specific probe ruled out the possibility that additional copies of *MtNIP3* were responsible for the increased expression of this gene in the leaves of B-tolerant lines. In fact, Caliph only had one gene copy as opposed to the two detected in Paraggio and *tap*. In barley, it was shown that a B-tolerant genotype had approximately four times as many copies of the B transporter gene *Bot1* compared to a number of intolerant genotypes [9], however, in Medicago this does not appear to be the case for *MtNIP3*.

With *MtNIP5* not showing measurable transcript levels in roots and youngest leaves, our findings suggest that *MtNIP3* is the most promising candidate to be involved in the mechanism controlling B toxicity tolerance in *M*. *truncatula* as its expression is strongly linked with B tolerance and distribution in the plant. Given that B is essential for cell wall elongation, where it is required for the cross-linking of the pectic polysaccharide rhamnogalacturonan II [24], the constitutive expression of *MtNIP3* in the roots and leaves is not surprising as MtNIP3 may always be needed to give B access to sink tissues for normal plant growth. The lower constitutive and then non-differential *MtNIP3* expression observed in the roots following B treatment is consistent with what was reported for *AtNIP6;1*, the Arabidopsis gene most similar to *MtNIP3*[20]. It would be interesting to know if this closest Medicago match to *AtNIP6*;1 does share the water impermeability of the AtNIP6;1 protein [20,25]. Considering that *M. truncatula* grows in low rainfall environments of northern Africa, Mediterranean Europe and southern Australia, a water conserving mechanism to remove excess B would be advantageous to the plant. It is likely that additional *MtNIP3* alleles, homologous or orthologous genes to the efflux transporter genes *AtBOR1*[12] or *HvBot1*[9,11] control B toxicity tolerance in other *M. truncatula* lines than the ones investigated here. The information gained in *M. truncatula* could also be used to facilitate the identification of genes in other pasture legumes (e.g. other Medicago species [6]) and also grain legumes (lentils [26], peas [6]) as recently demonstrated for alfalfa and the resistance to the fungal disease anthracnose [27].

The generated molecular markers are currently being used in SARDI’s pasture breeding program to confirm that B tolerance is conserved in advanced lines that also contain tolerance to the sulfonylurea family of herbicides [28], which is being selected for by using the gene-specific marker CAng [18]. These breeding aims support a crop/pasture rotation system where self-regenerating annual medics are grown in rotation with wheat and barley to fix atmospheric nitrogen to lower input costs, provide a cereal pathogen disease-break and serve as a high protein feed for livestock. At an estimated rate of approximately 25 kg nitrogen per tonne DM and a production of 4 tonnes DM per hectare, a medic crop can fix approximately 100 kg nitrogen per hectare [29], reducing the need for expensive inorganic fertilisers and at the same time avoiding CO_2_ emissions that would arise from the methane-driven fertiliser production.

## Conclusions

In the present study, a candidate gene approach was applied to establish a genetic linkage to the B toxicity tolerance trait observed in different cultivars of *Medicago truncatula*. Whereas no genetic linkage between other Boron exclusion and efflux transporter genes was detected, strong linkages were evident to genetic markers flanking a cluster comprising five predicted *NIP* genes explaining most (95%) of the phenotypic variation observed in the segregating Medicago populations. The derived markers are valuable tools for selection of this trait in a pasture breeding program and the close correlation of biomass and B toxicity qualifies B toxicity tolerance as a priority trait. The close correlation of *MtNIP3* expression and B concentration in roots and leaves of tolerant and intolerant lines suggests that this member of the gene cluster is the most promising candidate for a B tolerance gene in this species. It further suggests that the molecular basis of B detoxification in Medicago differs from that observed in the monocot barley where differential expression of *HvNIP2;1* in the roots [10] and a greater Boron-efflux transporter gene activity in the leaves [9] has previously been identified as the molecular basis of B toxicity alleviation. Further physiological studies on guttation and B leaching through rain, as recently described for barley [22] are required to quantify the possible role of these two mechanisms in mediating B toxicity tolerance.

## Methods

### Genetic material

Two F_2_ populations of *M*. *truncatula* were developed by crossing two B toxicity tolerant cultivars, Caliph and Paraggio (Caliph tested as Z-602 [6]), with the B toxicity intolerant line, *tap*. Paraggio and Caliph are cultivars developed by the South Australian Research and Development Institute (SARDI) and were registered as cultivars in 1982 and 1993, respectively, whereas *tap* (*mtapetala*) is a male sterile mutant derived from EMS treatment of A17 (Jemalong A17), the genotype used for *M. truncatula* genome sequencing [30]. For determination of the B tolerance trait inheritance, 316 and 313 F_2_ plants were assessed from the *tap* x Paraggio (*t*xP) and *tap* x Caliph (*t*xC) crosses, respectively, whereas 200 and 36 F_2_ plants from the *t*xP and *t*xC crosses, respectively, were used for molecular marker analysis.

### Plant growth conditions

Seeds from the three parental lines and the F_2_ progeny were surface sterilised, scarified with a small nick to the seedcoat with a scalpel blade, and allowed to germinate on moist filter paper in an inverted position. When the radicals had reached approximately 10 mm in length seeds were transferred onto a wire mesh and suspended over a 32 x 45 cm tub containing 10 L of a modified version of Hoagland’s nutrient solution (2 mM KNO_3_, 2 mM Ca(NO_3_)_2_.4H_2_O, 0.8 mM MgSO_4_.7H_2_O, 0.4 mM KH_2_PO_4_, and 0.036 mM NaFe(iii).EDTA of macronutrients, and 18.50 μM H_3_BO_3_, 3.66 μM MnCl_2_.4H_2_O, 0.31 μM ZnS0_4_.7H_2_O, 0.41 μM Na_2_MoO_4_.2H_2_O, and 0.13 μM CuSO_4_.5H_2_O of micronutrients, at pH 6.0). One 32 x 45 cm tub was sufficient to grow up to 200 plants. The nutrient solution was continually mixed with an aquarium pump and replaced weekly. The plants were grown in a glasshouse under natural light (12 hr day, 12 hr night) for 3 weeks and then treated with excess B by supplementing the aforementioned nutrient solution with 1 mM H_3_BO_3_.

### Quantification of boron tolerance

Visual phenotyping was performed 3 weeks after B treatment when the three oldest trifoliate leaves (of about 10–15 leaves) of each plant were scored using the following 0–5 scale: 0 = green leaf, 1 = slight chlorosis, 2 ≥ 10% leaf chlorosis, 3 = slight necrosis, 4 = 10-20% necrosis, 5 > 20% necrosis. The three leaves per plant were then harvested, pooled, and dried in a 65°C oven overnight.

The dried leaf samples from parental lines and a subset of approximately 40 highly B toxicity tolerant and intolerant F_2_ progeny from each cross were subjected to ICP-OES analysis [31] to measure their B concentrations. In an independent hydroponics experiment, root and leaf samples from the two tolerant and the intolerant parents, four plants each, were separately harvested in the manner as described above for ICP analysis. The wet roots were not rinsed but only blotted between paper towels to avoid B leaching as previously described [11]. The youngest two leaves were harvested to extract high quality genomic DNA for genotypic analysis.

### Quantification of plant biomass

The weight of total plant dry matter [mg plant^-1^] was measured from plants under high B stress in the *t*xC F_2_ population using 137 F_2_ lines and 5–9 repeats of their corresponding parents. Visual leaf symptom scores were taken before harvest, eight weeks after B treatment when the plants were a total of 11 weeks old.

### Development and detection of DNA markers

DNA was extracted from freeze-dried leaf tissue using a previously documented protocol [32] with modifications as described [33]. To identify *M*. *truncatula* homologues of *AtBOR1* and *AtNIP5;1*, their corresponding cDNA (GenBank: AB073713 and NM_117106) and protein (GenBank: BAC20173 and NP_192776) sequences were used to query the *M*. *truncatula* pseudomolecule database, version Mt3.5 (http://www.medicagohapmap.org). Once identified, nearby SSR markers were screened by PCR on a subset of lines from both *t*xP and *t*xC F_2_ populations to establish linkage. PCRs with the SSR primers were carried out in 12.5 μL reactions containing 1X reaction buffer (QIAGEN, Hilden, Germany), 1X Q solution (QIAGEN), 0.16 mM MgCl_2_, 0.32 μM of each primer, 0.4 U of Taq DNA polymerase (QIAGEN) and approximately 50 ng of DNA as template. The PCRs were effected using the following cycling conditions: 90 s at 95°C, then 35 cycles of 20 s at 95°C, 30 s at 58°C, and 30 s at 72°C, and a final extension step of 5 min at 72°C. All PCR products were electrophoresed on 8% polyacrylamide gels which were stained with ethidium bromide and visualised under UV light.

For CAPS (cleaved amplified polymorphic sequence) marker development, PCR primers were designed on the sequence of the A17 genotype. Genomic regions were amplified by PCR in 12.5 μL reactions as described earlier but with a 72°C extension step ranging from 30–90 s depending on the expected size of the amplicon. PCR products were resolved on 1.2% agarose gels and sequenced using the Big Dye Terminator Version 3.1 labelling kit (Applied Biosystems, Foster City, CA) according to the manufacturer’s protocol but using one-eighth of the recommended reaction mix. DNA sequencing was performed on an ABI 3730 DNA Sequencer (Applied Biosystems) and DNA sequences were analysed using Vector NTI software (Invitrogen). The SNP2CAPS program [34] was then used to develop CAPS markers from the sequence alignments. PCR products were digested with the relevant restriction enzymes according to the manufacturer’s protocol and separated on 1.5% agarose gels.

Southern hybridization of parental DNA was carried out according to standard protocols [35] using 10 μg of digested DNA per lane and the restriction enzymes *Bam*HI, *Dra*I, *Eco*RI, *Eco*RV, *Hin*dIII and *Xba*I (5 U/μg).

### Analysis of candidate B transporter gene expression

To monitor the expression of candidate B transporter genes, plants were grown in a controlled-environment room for 3 weeks using the supported hydroponics system previously described [36] but with the same nutrient solution as described earlier. Whole roots and the three youngest trifoliates from each plant were harvested and frozen in liquid nitrogen immediately before supplementing the nutrient solution with 1 mM H_3_BO_3_ (+B) and then at 3, 6, 12, 24, and 48 hr after B treatment. A control set of plants was allowed to grow for 3 weeks after B treatment to ensure that adequate symptoms developed.

Total RNA was extracted from frozen root and young leaf tissue using TRI Reagent (Sigma-Aldrich, St. Louis, MO). For each time point, RNA was extracted from three independent biological replicates which were pooled to average out biological variability. To prepare cDNA template for quantitative real-time RT-PCR (Q-PCR), RNA was first treated with 2 U RNase-free *DNase* I (Ambion, Austin, TX) to remove residual DNA. cDNA was then synthesised from 3 μg of RNA using 200 U of SuperScript III reverse transcriptase (Invitrogen) according to the manufacturer’s instructions with quality being confirmed via PCR detection of transcripts from the gene *actin* using earlier described primers [37]. Q-PCR was performed in an RG 3000 Rotor-Gene Real Time Thermal Cycler (Corbett Research, Sydney, Australia). The final reaction volume of 8 μL comprised a mixture of 1X Sensimix SYBR green PCR reagent (Bioline), 0.4 μM of each forward and reverse gene-specific primer, and 3 μL of a 1:20 dilution of cDNA. The PCR was effected using the following cycling conditions, 10 min at 95°C, followed by 35 cycles of 20 s at 95°C, 30 s at 58°C and 30 s at 72°C. All Q-PCR reactions were carried out in duplicate and melt curve analysis was performed at the end of each run to confirm that there was no signal from non-specific binding products. No-template controls were also included in each run to test for possible contamination of assay reagents. The sequences of all target gene primers and the reference gene *actin* primers are shown in additional file 3.

To quantify gene expression levels, DNA standards were generated by cloning each target and reference gene PCR product into the pDrive cloning vector (QIAGEN) according to the manufacturer’s instructions. After spectrophotometric determination of plasmid DNA concentration, the vector was linearised with the restriction enzyme *Nru*I, and the copy number of standard DNA molecules calculated using the formula (*x* g/μL DNA / (plasmid length in base pairs × 660)) × 6.022 × 10^23^ = *y* molecules/μL. Serial ten-fold dilutions of a given DNA standard (10^7^-10^1^ copies/ μL) were then incorporated into each run to quantify the number of target gene transcripts. These values were then normalised against the quantities of transcripts detected from the reference gene *actin*.

## Abbreviations

B: Boron; Mt: *Medicago truncatula*; T: tap; C: Caliph; P: Paraggio; ICP spectrometry: Inductively coupled plasma spectrometry; MIP: Major intrinsic protein; SSR marker: Single sequence repeat marker; CAPS marker: Cleaved amplified polymorphic sequence marker.

## Competing interests

The authors declare that they have no competing interest.

## Authors’ contribution

PB performed most of the experiments, was involved in experimental design, and drafted the manuscript, DMP prepared the crosses of the segregating populations and was involved in the setup of the hydroponics system, RMN was involved in the conceptualisation of the project, JH helped with the selection of the population parents, KHO conceptualised the idea, was involved in experimental design and coordination, and drafted the manuscript. All authors read and approved the final manuscript.

## Supplementary Material

Additional file 1 ***MtNIP3 *cDNA sequence alignments for parental lines Caliph, Paraggio, and the intolerant *tap*.** Single nucleotide polymorphisms between sequences are highlighted. Click here for file

Additional file 2 **MtNIP3 protein sequence alignments for parental lines Caliph, Paraggio, and the intolerant *tap*.** Amino acid differences between sequences are highlighted. Click here for file

Additional file 3 Q-PCR primers, primers for Southern probe and promoter analysis. Click here for file
