# Supplementary material for: Genetic analysis of tolerance to Boron toxicity in the legume Medicago truncatula
Source: BMC Plant Biol. 2013 Mar 27;13:54. doi: 10.1186/1471-2229-13-54 (PMC3636127; doi:10.1186/1471-2229-13-54)
Supplement: Additional file 1 — MtNIP3 cDNA sequence alignments for parental lines Caliph, Paraggio, and the intolerant tap. Single nucleotide polymorphisms between sequences are highlighted. [file 1471-2229-13-54-S1.pdf]

```
            1                                                         60
Caliph      ATGGACAATGAGGAAATCCCATCAGTACCTTCTACCCCTGCAACACCAGGTACTCCTGGT
Paraggio    ATGGACAATGAGGAAATCCCATCAGTACCTTCTACCCCTGCAACACCAGGTACTCCTGGT
tap         ATGGACAATGAGGAAATCCCATCAGTACCTTCTACCCCTGCAACACCAGGTACTCCTGGT

            61                                   *                   120
Caliph      GCTCCTCTTTTTGGTGGCTTCAGGTCTGAGAGAACTGGAAATGGTAGAAAGAACTCACTC
Paraggio    GCTCCTCTTTTTGGTGGCTTCAGGTCTGAGAGAACTGAAAATGGTAGAAAGAACTCACTC
tap         GCTCCTCTTTTTGGTGGCTTCAGGTCTGAGAGAACTGGAAATGGTAGAAAGAACTCACTC

            121                                                      180
Caliph      CTCAAGAATTTAAAATGTTTTAGTGTTGAAGATTGGACTTTGGAAGATGGAGCCTTACCT
Paraggio    CTCAAGAATTTAAAATGTTTTAGTGTTGAAGATTGGACTTTGGAAGATGGAGCCTTACCT
tap         CTCAAGAATTTAAAATGTTTTAGTGTTGAAGATTGGACTTTGGAAGATGGAGCCTTACCT

            181                                                      240
Caliph      AAAGTCACTTGCTCTTTACCTCCCCCTCCTGTCCCTCTTGCCAAAAAGGTTGGAGCTGAG
Paraggio    AAAGTCACTTGCTCTTTACCTCCCCCTCCTGTCCCTCTTGCCAAAAAGGTTGGAGCTGAG
tap         AAAGTCACTTGCTCTTTACCTCCCCCTCCTGTCCCTCTTGCCAAAAAGGTTGGAGCTGAG

            241        *       *                                     300
Caliph      TTTATAGGCACGTACATTCTAATGTTTGCTGGAATAGCCACTGCAATTGTGAACCAAAAG
Paraggio    TTTATAGGCACATACATTGTAATGTTTGCTGGAATAGCCACTGCAATTGTGAACCAAAAG
tap         TTTATAGGCACATACATTCTAATGTTTGCTGGAATAGCCACTGCAATTGTGAACCAAAAG

            301                                                      360
Caliph      ATACATAACTCAGAAACACTAATTGGATGTGCTGGAGCAACTGGACTTGCTGTTATGATC
Paraggio    ATACATAACTCAGAAACACTAATTGGATGTGCTGGAGCAACTGGACTTGCTGTTATGATC
tap         ATACATAACTCAGAAACACTAATTGGATGTGCTGGAGCAACTGGACTTGCTGTTATGATC

            361                                                      420
Caliph      ATAATATTATCAACCGGTCACATTTCCGGTGCTCATCTTAATCCGGCTGTCACGATTTCA
Paraggio    ATAATATTATCAACCGGTCACATTTCCGGTGCTCATCTTAATCCGGCTGTCACGATTTCA
tap         ATAATATTATCAACCGGTCACATTTCCGGTGCTCATCTTAATCCGGCTGTCACGATTTCA

            421                                      *               480
Caliph      TTTGCTGCATTAAAACACTTCCCCTGGAAAAATGTACCTTTATATATTGCTGCACAAGTT
Paraggio    TTTGCTGCATTAAAACACTTCCCCTGGAAAAATGTACCTTTGTATATTGCTGCACAAGTT
tap         TTTGCTGCATTAAAACACTTCCCCTGGAAAAATGTACCTTTGTATATTGCTGCACAAGTT

            481                                                      540
Caliph      TTGGCATCAATATGTGCTTCATTTACTTTGAAAGGGGTTTTTCATCCATTTATGAGTGGT
Paraggio    TTGGCATCAATATGTGCTTCATTTACTTTGAAAGGGGTTTTTCATCCATTTATGAGTGGT
tap         TTGGCATCAATATGTGCTTCATTTACTTTGAAAGGGGTTTTTCATCCATTTATGAGTGGT

            541                                                  *   600
Caliph      GGTGTCACGGTTCCTTCAGTTGAATATGGCCAAGCTTTTGCTTTAGAGTTTATCATCAGC
Paraggio    GGTGTCACGGTTCCTTCAGTTGAATATGGCCAAGCTTTTGCTTTAGAGTTTATTATCAGC
tap         GGTGTCACGGTTCCTTCAGTTGAATATGGCCAAGCTTTTGCTTTAGAGTTTATCATCAGC
```

```
            601                                                        660
Caliph      TTTAATCTCATGTTTGTTGTCACTGCAGTGGCCACCGACACAAGAGCTGTGGGAGAACTT
Paraggio    TTTAATCTCATGTTTGTTGTCACTGCAGTGGCCACCGACACAAGAGCTGTGGGAGAACTT
tap         TTTAATCTCATGTTTGTTGTCACTGCAGTGGCCACCGACACAAGAGCTGTGGGAGAACTT

            661         *                                              720
Caliph      GCGGGAATCGCGGTGGGAGCCACTGTCATGCTCAACATACTCATTGCTGGGCCTGCAACT
Paraggio    GCGGGAATCGCAGTGGGAGCCACTGTCATGCTCAACATACTCATTGCTGGGCCTGCAACT
tap         GCGGGAATCGCGGTGGGAGCCACTGTCATGCTCAACATACTCATTGCTGGGCCTGCAACT

            721                                                        780
Caliph      GGAGCTTCAATGAATCCAGTAAGAACATTAGGACCAGCAATTGCTGCAAACAACTACAAA
Paraggio    GGAGCTTCAATGAATCCAGTAAGAACATTAGGACCAGCAATTGCTGCAAACAACTACAAA
tap         GGAGCTTCAATGAATCCAGTAAGAACATTAGGACCAGCAATTGCTGCAAACAACTACAAA

            781                                                        840
Caliph      GGCATATGGTTATATCTCATAGCCCCTATTCTTGGTGCCCTTGGTGGGGCAGGTGCTTAC
Paraggio    GGCATATGGTTATATCTCATAGCCCCTATTCTTGGTGCCCTTGGTGGGGCAGGTGCTTAC
tap         GGCATATGGTTATATCTCATAGCCCCTATTCTTGGTGCCCTTGGTGGGGCAGGTGCTTAC

            841 *                                                      900
Caliph      ACTGTTGTTAAGCTTCCTGATGAAGAGTTTAACTCTGAGGTAAAAGCCTCTTCTGCCCCT
Paraggio    ACTGCTGTTAAGCTTCCTGATGAAGAGTTTAACTCTGAGGTAAAAGCCTCTTCTGCCCCT
tap         ACTGCTGTTAAGCTTCCTGATGAAGAGTTTAACTCTGAGGTAAAAGCCTCTTCTGCCCCT

            901             918
Caliph      GGAAGTTTCAGAAGATGA
Paraggio    GGAAGTTTCAGAAGATGA
tap         GGAAGTTTCAGAAGATGA
```

Additional file 1
