# Supplementary material for: Genetic analysis of tolerance to Boron toxicity in the legume Medicago truncatula
Source: BMC Plant Biol. 2013 Mar 27;13:54. doi: 10.1186/1471-2229-13-54 (PMC3636127; doi:10.1186/1471-2229-13-54)
Supplement: Additional file 2 — MtNIP3 protein sequence alignments for parental lines Caliph, Paraggio, and the intolerant tap. Amino acid differences between sequences are highlighted. [file 1471-2229-13-54-S2.pdf]

```
                1                               *                          60
Caliph          MDNEEIPSVPSTPATPGTPGAPLFGGFRSERTGNGRKNSLLKNLKCFSVEDWTLEDGALP
Paraggio        MDNEEIPSVPSTPATPGTPGAPLFGGFRSERTENGRKNSLLKNLKCFSVEDWTLEDGALP
tap             MDNEEIPSVPSTPATPGTPGAPLFGGFRSERTGNGRKNSLLKNLKCFSVEDWTLEDGALP

                61                        *                         120
Caliph          KVTCSLPPPPVPLAKKVGAEFIGTYILMFAGIATAIVNQKIHNSETLIGCAGATGLAVMI
Paraggio        KVTCSLPPPPVPLAKKVGAEFIGTYIVMFAGIATAIVNQKIHNSETLIGCAGATGLAVMI
tap             KVTCSLPPPPVPLAKKVGAEFIGTYILMFAGIATAIVNQKIHNSETLIGCAGATGLAVMI

                121                                                        180
Caliph          IILSTGHISGAHLNPAVTISFAALKHFPWKNVPLYIAAQVLASICASFTLKGVFHPFMSG
Paraggio        IILSTGHISGAHLNPAVTISFAALKHFPWKNVPLYIAAQVLASICASFTLKGVFHPFMSG
tap             IILSTGHISGAHLNPAVTISFAALKHFPWKNVPLYIAAQVLASICASFTLKGVFHPFMSG

                181                                                        240
Caliph          GVTVPSVEYGQAFALEFIISFNLMFVVTAVATDTRAVGELAGIAVGATVMLNILIAGPAT
Paraggio        GVTVPSVEYGQAFALEFIISFNLMFVVTAVATDTRAVGELAGIAVGATVMLNILIAGPAT
tap             GVTVPSVEYGQAFALEFIISFNLMFVVTAVATDTRAVGELAGIAVGATVMLNILIAGPAT

                241                                       *                300
Caliph          GASMNPVRTLGPAIAANNYKGIWLYLIAPILGALGGAGAYTVVKLPDEEFNSEVKASSAP
Paraggio        GASMNPVRTLGPAIAANNYKGIWLYLIAPILGALGGAGAYTAVKLPDEEFNSEVKASSAP
tap             GASMNPVRTLGPAIAANNYKGIWLYLIAPILGALGGAGAYTAVKLPDEEFNSEVKASSAP

                301 305
Caliph          GSFRR-
Paraggio        GSFRR-
tap             GSFRR-
```

Additional file 2
