# Supplementary material for: Genetic analysis of tolerance to Boron toxicity in the legume Medicago truncatula
Source: BMC Plant Biol. 2013 Mar 27;13:54. doi: 10.1186/1471-2229-13-54 (PMC3636127; doi:10.1186/1471-2229-13-54)
Supplement: Additional file 3 — Q-PCR primers, primers for Southern probe and promoter analysis. [file 1471-2229-13-54-S3.pdf]

Gene	Primer sequence (forward and reverse, 5' – 3')	Expected fragment size [bp] cDNA	gDNA
Medtr4g006630.1	CGTTTTTCAAAATCTGGTTGG<br>TGAGCACCAGAAATGTGACC	183	no product
Medtr4g006630.1 (2)	TTTTCGTAAAGGTTTCAAAGTGT<br>ATGCAGCAAATGAAATCGTG	243	612
Medtr4g006650.1*	AAGAGAAGGCTGCACCTGAA (for)<br>CCTCTGCACAAGCTTCACAA (rev1)<br>TTCAGTTGAATATGGCCAAGC (rev2)	96/194	198
Medtr4g006660.1	GGTTGGAGCTGAGTTTATAGGC<br>CCTCCAAGTCCTTCATTTGC	218	218
Medtr4g006670.1	TGGTGCTCTACATAAGTTCCAATC<br>GGGGCAGAAGAGGCTTTTAC	108	319
Medtr4g006730.1**	GGTTGGAGCTGAGTTTATAGGC<br>TTTTTCCAGGGGAAGTGTTTT	225	225
Medtr4g006730.2	AGAGCTGTGGGAGAACTTGC<br>TGAGGAGTGAAAGTTGGAACA	92	no product
MtActin	ATGTTGCTATTCAGGCCG<br>GCTCATAGTCAAGGGCAAT	Previously described [34]	
Mt730probe	TGATTAGAGTATGGACAATGAGGAA<br>CTTTTTGGCAAGAGGGACAG	-	238
Mt730promoter	CCACTTTACGCCACTTAACG<br>GCTCCATCTTCCAAAGTCCA	-	1304

*Also specific for similar gene on chromosome 3;
**Also specific for 630.1 and 730.2.

Additional file 3
